# Beta tACS of varying intensities differentially affect resting-state and movement-related M1-M1 connectivity

**DOI:** 10.3389/fnins.2024.1425527

**Published:** 2024-09-20

**Authors:** Kym Wansbrough, Welber Marinovic, Hakuei Fujiyama, Ann-Maree Vallence

**Affiliations:** ^1^School of Psychology, College of Health and Education, Murdoch University, Perth, WA, Australia; ^2^Centre for Healthy Ageing, Health Futures Institute, Murdoch University, Perth, WA, Australia; ^3^Centre for Molecular Medicine and Innovative Therapeutics, Health Futures Institute, Murdoch University, Perth, WA, Australia; ^4^School of Population Health, Curtin University, Perth, WA, Australia

**Keywords:** transcranial alternating current stimulation, electroencephalography, neural oscillations, beta oscillations, motor cortex, motor control, connectivity

## Abstract

Due to the interconnected nature of the brain, changes in one region are likely to affect other structurally and functionally connected regions. Emerging evidence indicates that single-site transcranial alternating current stimulation (tACS) can modulate functional connectivity between stimulated and interconnected unstimulated brain regions. However, our understanding of the network response to tACS is incomplete. Here, we investigated the effect of beta tACS of different intensities on phase-based connectivity between the left and right primary motor cortices in 21 healthy young adults (13 female; mean age 24.30 ± 4.84 years). Participants underwent four sessions of 20 min of 20 Hz tACS of varying intensities (sham, 0.5 mA, 1.0 mA, or 1.5 mA) applied to the left primary motor cortex at rest. We recorded resting-state and event-related electroencephalography (EEG) before and after tACS, analyzing changes in sensorimotor beta (13–30 Hz) imaginary coherence (ImCoh), an index of functional connectivity. Event-related EEG captured movement-related beta activity as participants performed self-paced button presses using their right index finger. For resting-state connectivity, we observed intensity-dependent changes in beta ImCoh: sham and 0.5 mA stimulation resulted in an increase in beta ImCoh, while 1.0 mA and 1.5 mA stimulation decreased beta ImCoh. For event-related connectivity, 1.5 mA stimulation decreased broadband ImCoh (4–90 Hz) during movement execution. None of the other stimulation intensities significantly modulated event-related ImCoh during movement preparation, execution, or termination. Interestingly, changes in ImCoh during movement preparation following 1.0 mA and 1.5 mA stimulation were significantly associated with participants’ pre-tACS peak beta frequency, suggesting that the alignment of stimulation frequency and peak beta frequency affected the extent of neuromodulation. Collectively, these results suggest that beta tACS applied to a single site influences connectivity within the motor network in a manner that depends on the intensity and frequency of stimulation. These findings have significant implications for both research and clinical applications.

## 1 Introduction

Over the past decade, there has been growing interest in the use of transcranial alternating current stimulation (tACS) for neuromodulation. tACS is a safe and painless non-invasive brain stimulation technique, which delivers a weak sinusoidal electrical current through surface electrodes placed on the scalp (Antal et al., 2008). Evidence suggests that tACS can modulate various cognitive and behavioral processes, such as attentional control and motor learning (for a review, see Klink et al., 2020), likely by entraining the neural oscillations that underpin these processes (Wischnewski et al., 2023). Through this neuromodulation, tACS offers a unique avenue for advancing knowledge regarding the neural correlates of various cognitive and motor functions, and holds great promise as a therapeutic intervention for individuals with neurological and psychiatric disorders. Currently, the potential of tACS is constrained by our incomplete understanding of its neurophysiological effects. In recent years, we have gained substantial insights into the tACS response at or near the stimulation site: the modulation of neural oscillatory power is likely underpinned by changes in neural spike timing and metabolic activity (for a review of tACS mechanisms, see Wischnewski et al., 2023). However, less is known about the effect of tACS beyond the stimulation site.

The brain comprises networks of structurally and functionally connected regions, which interact to produce cognition and behavior (Jarrett, 2011; Power et al., 2011; Bassett and Sporns, 2017). Through these connections, tACS might induce neural changes in unstimulated brain regions of the connectome. Indeed, following single-site (i.e., unifocal) tACS, there have been changes at the intra-hemispheric, inter-hemispheric, and global levels (e.g., Alekseichuk et al., 2016; Fuscà et al., 2018; Schwab et al., 2019; Wessel et al., 2020; Hosseinian et al., 2021; Clancy et al., 2022; Peng et al., 2023). While we have garnered some insights into the network effects of tACS, our understanding remains incomplete, particularly within the motor system. Within the motor system, there is a complex network of neural pathways that enables coordinated ommunication between the left and right primary motor cortices (M1s; Ruddy et al., 2017). This network is essential for producing smooth, precise, and coordinated movement (Guye et al., 2003; Stagg et al., 2014). Importantly, communication through coherence theory (Fries, 2005, 2015) suggests the phase-dependent synchronization of neural oscillations is fundamental for transmitting information efficiently between between M1s.

Motor cortical beta oscillations (13–30 Hz) have been suggested to play a fundamental role in movement, as they show a robust pattern of movement-related changes: (1) pre-movement beta event-related desynchronization (ERD), (2) movement beta ERD, and (3) post-movement beta event-related synchronization (ERS; for a review, see Kilavik et al., 2013). The beta ERD is thought to reflect the activation of motor areas for movement preparation and execution, and the movement ERS is thought to reflect motor inhibition (Kilavik et al., 2013). When applied unifocally to the left M1, beta tACS has been shown to induce changes in motor neurophysiology (e.g., corticospinal excitability; Wischnewski et al., 2019) and motor behavior (e.g., motor learning; Krause et al., 2016; Pollok et al., 2015; Yamaguchi et al., 2020). Weinrich et al. (2017) investigated the network effects of left M1 beta tACS, by examining the changes in blood-oxygen-level-dependent (BOLD) activity of left and right M1s, as well as the premotor cortices (PMCs). Functional magnetic resonance imaging (fMRI) scans were performed during 20 Hz, 5 Hz (control frequency), or sham stimulation, applied to the left M1. A positive relationship between M1-M1 connectivity and overall motor network strength (indexed by inter-regional BOLD correlations) was observed during 5 Hz and sham stimulation. Notably, this relationship was not present during 20 Hz tACS, indicating that the stimulation modulated motor network connectivity in a frequency-specific manner, with beta tACS shifting the phase of the stimulated left M1 away from the resonant phase of the non-stimulated motor regions. However, this phase shift theory could not be tested by analyzing BOLD activity, as this measure does not capture the phase alignment of inter-regional neural activity.

Post-tACS changes in phase-based connectivity were examined by Wischnewski et al. (2019), using electroencephalography (EEG). Applying 15 min of beta (20 Hz) tACS at 1.0 mA to left M1 did not significantly modulate M1-M1 beta connectivity. However, as the primary aim of their study was to examine the effect of dextromethorphan (an *N*-methyl-D-aspartate receptor antagonist) on the tACS response, their design included a placebo with active tACS but not a sham control. Thus, when applying unifocal stimulation to left M1, the effect of real vs. sham tACS on phased-based M1-M1 connectivity remains unclear. Furthermore, it is possible that the stimulation intensity might differentially modulate phase-based M1-M1 connectivity. Dynamic systems theory (Pikovsky et al., 2001) suggests that the stimulation intensity would affect tACS-induced entrainment in a positive and linear manner, which has been supported by evidence from *in vivo* animal models of rodents and primates (Asan et al., 2020; Huang et al., 2021; Johnson et al., 2020; Krause et al., 2022). In humans, intensity-response relationships have been examined in post-tACS corticospinal excitability (Moliadze et al., 2012; Shorafa et al., 2021) and oscillatory power (De Koninck et al., 2021; Wang et al., 2022). These findings have been mixed: some studies have observed a positive and linear intensity-response relationship (Moliadze et al., 2012), while others have not (De Koninck et al., 2021; Shorafa et al., 2021; Wang et al., 2022). Additionally, the effect of varying beta tACS intensity on M1-M1 connectivity remains unknown.

In the current study, we examined the effect of different intensities of unifocal beta tACS on both resting-state and event-related M1-M1 EEG connectivity in healthy young adult humans. Event-related EEG recordings captured movement-related beta activity, elicited by participants performing self-paced voluntary hand movements. High definition tACS was applied to the hand-area of the left M1 at varying intensities (0.5 mA, 1.0 mA, 1.5 mA), and sham stimulation was included as a control. Changes in both resting-state and event-related connectivity were examined, as each state provides unique and functionally valuable insights into the brain’s dynamic activity at baseline during specific motor processes (Wu et al., 2014; Yamaguchi et al., 2020; Cheng et al., 2021; Titone et al., 2022). In line with the phase shift theory, posited by Weinrich et al. (2017), we expected that M1-M1 connectivity would decrease following unifocal beta tACS. Based on dynamic systems theory and findings from online evaluation of tACS intensity within *in vivo* animal models (vs. the offline evaluation in human models), it was hypothesized that beta tACS would linearly decrease resting-state and event-related beta connectivity.

## 2 Materials and methods

### 2.1 Participants

Forty-seven healthy young adults were originally recruited to attend four experimental sessions. Of those, 24 participants did not complete all four sessions: 4 attended three sessions, 10 attended two sessions, and 10 attended one session before dropping out due to unforeseen personal circumstances (*n* = 14), COVID-19 lockdown (*n* = 8), or minor adverse events (1 experienced a headache and 1 experienced dizziness). A total of 23 healthy young adults participated in all four experimental sessions (13 female; age range = 18 to 34 years; mean age = 24.30 ± 4.84 years). Participants were right-handed, as assessed by the Edinburgh Handedness Inventory (Oldfield, 1971; range = 41.18–100.00; M = 87.59, SD = 16.22), had no contraindications to non-invasive brain stimulation (Rossini et al., 1994; Rossi et al., 2009), and had no history of neurological conditions. All participants provided written informed consent. The experiment was conducted in accordance with the Declaration of Helsinki and was approved by the Murdoch University Human Research Ethics Committee (2018/098).

### 2.2 Experimental design

The experiment was a sham controlled, triple-blinded, within-subjects design. Each participant completed four sessions, separated by at least 72 h (mean inter-session interval = 11.68 ± 6.46 days; Chaieb et al., 2014; Stecher et al., 2017; Saito et al., 2022). The independent variable was stimulation intensity: sham, 0.5 mA, 1.0 mA, and 1.5 mA. The order of stimulation intensities was counterbalanced across participants. Individual participants were tested at the same time of day so that inter-session differences in post-tACS ImCoh could not be attributed to the time of testing (Wilson et al., 2014). Participants were unaware of the intensities applied in each session, being only informed that variations in stimulation settings were being investigated, including a sham session. At the end of the fourth session, it was revealed that each session varied in stimulation intensity. The researcher who conducted data collection and analysis (KW) was blinded to the stimulation intensities. An independent researcher (AMV) pre-set the stimulation parameters and randomly assigned each intensity to a label: ‘A,’ ‘B,’ ‘C,’ or ‘D.’ The researcher conducting the analysis (KW) remained blinded to the conditions until all analyses were completed.

### 2.3 Transcranial alternating current stimulation (tACS)

High-definition tACS (HD-tACS) was delivered through conductive round rubber electrodes (2 cm diameter; 3.14 cm^2^ area) via a neuroConn DC-STIMULATOR MC (NeuroConn, Ilmenau, Germany). To reduce impedance, a Ten20 conductive paste was placed between the surface of the electrodes and the scalp. Impedance was kept below 50 kΩ.

A 4 × 1 HD-tACS electrode montage was used, as it has been shown to deliver a more focal current to M1 than the standard bipolar tACS montage (Datta et al., 2008; Edwards et al., 2013). The center electrode was placed over the left M1 representation of the first-dorsal interosseous (FDI), which was located using transcranial magnetic stimulation (TMS; full details regarding TMS procedure provided in Supplementary Methods). Current densities for the center electrode were 0.159 mA/cm^2^ for 0.5 mA stimulation, 0.318 mA/cm^2^ for 1 mA stimulation, and 0.478 mA/cm^2^ for 1.5 mA stimulation. The four return electrodes were placed at a 50 mm radius from the center electrode. Placement of the return electrodes was based on electric current simulations in a model of the average adult head (MNI152; conducted with SimNIBS v3.2.0; Thielscher et al., 2015). Current densities for each of the return electrodes were 0.040 mA/cm^2^ for 0.5 mA stimulation, 0.080 mA/cm^2^ for 1 mA stimulation, and 0.120 mA/cm^2^ for 1.5 mA stimulation. Electric field models can be found in the Supplementary Methods.

Sinusoidal stimulation was delivered at 20 Hz with zero DC-offset, for 20 min. Both this stimulation frequency and duration have been shown to induce changes in neurophysiological measures (Neuling et al., 2013; Heise et al., 2016; e.g., resting-state EEG, corticospinal excitability and functional near-infrared spectroscopy; Berger et al., 2018) and behavior (e.g., motor learning, Pollok et al., 2015). For all real stimulations, there were a 30 s ramp up period to the target intensity and a 30 s ramp down period (Woods et al., 2016). For the sham stimulation, a 30 s ramp up was immediately followed by a 30 s ramp down at 0 and 20 min. For all participants, the sham tACS current ramped up to 1.5 mA (the highest stimulation intensity that was investigated in this experiment). Results from previous studies indicated that this sham tACS protocol was sufficient for eliciting the typical sensation usually perceived at the onset of active tACS (Gandiga et al., 2006; Ambrus et al., 2012; Woods et al., 2016).

### 2.4 Electroencephalography

EEG was collected with a 128-electrode EGI HydroCel™ Geodesic Sensor Net (Electrical Geodesics, Inc., Eugene, OR), following the international 10–20 system of electrode placement (Klem et al., 1999; Jurcak et al., 2007). EEG signals were acquired using EGI Net Amps 300 amplifiers and Netstation 4.5.6, band pass filtered (0.05 to 100 Hz), and digitised at a sampling rate of 1,000 Hz. Signals were referenced to Cz during recording, and impedance was kept below 50 kΩ (Nelson et al., 2017; Alhajri et al., 2018; Angelini et al., 2018; Tatti et al., 2019) as per the manufacturer’s recommendation (Magstim EGI, Eugene, OR). The HydroCel Geodesic Sensor Net allowed us to place the tACS electrodes without having to remove the EEG net.

#### 2.4.1 EEG recording procedure

Two types of EEG recordings were taken: (1) resting-state recordings; (2) event-related recordings. For the resting-state recordings, participants were instructed to keep their eyes open and look straight ahead at a fixation cross for 3 min. For the event-related recordings, participants were instructed to perform self-paced isometric flexions of the right index finger at approximately 10 s intervals. An index finger flexion was chosen, as it has been shown to elicit the three event-related changes in beta activity over the sensorimotor cortex (e.g., Rimbert et al., 2018). Movements were self-paced to engage the neural processes involved in generating internally motivated voluntary movements, which slightly differ from externally motivated movements (Haggard et al., 2006; e.g., reduced motor preparation; Brass and Haggard, 2008). The tip of the index finger was placed on an 8 mm 10 N/2.2 lb. SingleTact force sensor (SingleTact, Glasgow, UK) that was permanently secured on a computer mouse. Participants performed a total of 60 index finger flexions, which were split into two blocks of 30 flexions. Before the experimental session began, participants completed one 60 s finger flexion training block with a go signal (“press” presented on a screen ever 10 s) and one block that was self-paced, with verbal feedback from the experimenter. For the duration of event-related recordings, participants were instructed to look straight ahead at a fixation cross to avoid random eye movements, and minimize any other movement (e.g., blinking, swallowing, etc).

#### 2.4.2 Force sensor event trigger

As stated above, the event-related recordings required participants to press into a force sensor. This was done so that the onset of movement could be registered as a NetStation (Electrical Geodesics, Inc.) digital input event for data analysis. The force sensor output was digitized at a sampling value of 5,000 Hz (CED Power1401), data were acquired using Signal (version 6.02, Cambridge Electronic Design), and event-triggers were generated in NetStation at the onset of movement, using a custom-written Signal sequencer. The onset of movement was defined as the moment at which the force-trace exceeded a pre-determined threshold (which was determined for each participant during practice trials).

### 2.5 Procedure

**Figure 1:**
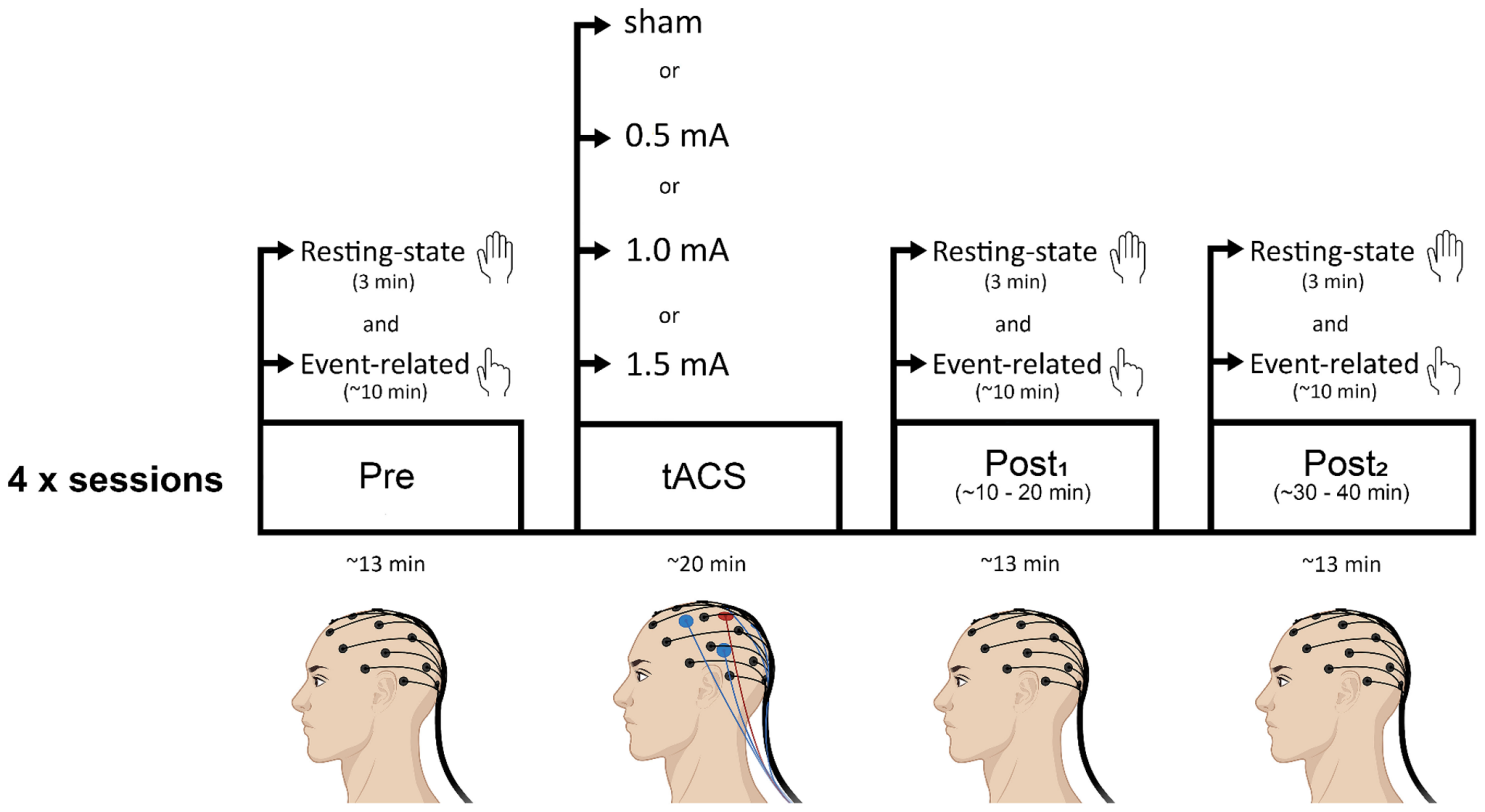
Schematic of the experimental timeline. Participants attended four sessions (each separated by a minimum of 72 h): three real tACS sessions in which intensity of stimulation was varied (0.5 mA, 1.0 mA, or 1.5 mA), and a sham (control) stimulation session. Sessions were counterbalanced across participants. Beta tACS was applied to the hand area of left M1 at 20 Hz for 20 min. Resting-state EEG and event-related EEG recordings were obtained before tACS (pre), and at two time points following tACS (post_1_, post_2_). Resting-state EEG recordings always preceded event-related EEG recordings.

Figure 1 shows a schematic of the experimental timeline. At the beginning of each session, the left M1 representation of the FDI was located for tACS electrode placement and the EEG cap was fitted. After this, baseline measures of resting-state and event-related EEG were recorded. Then, tACS electrodes were placed underneath the EEG net, onto the scalp, and either real or sham stimulation was delivered for 20 min. EEG was not recorded during the stimulation period. Following stimulation, the tACS electrodes were removed and both resting-state and event-related EEG were recorded (as per baseline measures) at two time-points: ~5 min following tACS (post_1_; range = 3–8 min post-tACS; mean time = 5.5 min post-tACS) and ~ 25 min following tACS (post_2_; range = 20–29.5 min post-tACS; mean time = 24.6 min post-tACS). Before each EEG recording block, experimenters ensured that impedance levels were below threshold. The delay between stimulation end and the first post-tACS recording was due to the time required to remove the tACS electrodes and bring impedance below threshold. Each session lasted approximately 2.5 h.

### 2.6 Tolerability and blinding

At the end of each session, participants completed a self-report questionnaire regarding perceived sensations and adverse effects induced by tACS (Fujiyama et al., 2023). Additionally, to monitor blinding effectiveness, participants were asked whether they thought they received real or sham tACS and their confidence on a scale of 1 (not confident at all) to 10 (very confident).

### 2.7 Data analysis

#### 2.7.1 EEG pre-processing

EEG data were pre-processed using the EEGLAB toolbox (Delorme and Makeig, 2004) through the MATLAB environment (MathWorks, R2020b). All EEG data were down-sampled to 500 Hz, bandpass filtered from 0.5 to 95 Hz, and notch filtered at 50 Hz. The data were then epoched: resting-state data were divided into 2,000 ms segments; event-related data were segmented from –2,500 to 4,500 ms relative to stimulus onset. Bad channels and noisy epochs were then visually identified and manually removed, and all removed channels were interpolated. The data were then re-referenced to the average, using the *fullRankAveRef* EEGLAB plugin. Next, independent component analysis (ICA) was performed, using the Infomax algorithm. Following ICA, components containing artifacts clearly distinguished from brain-driven EEG signals (e.g., ocular, vascular, and myogenic artifacts) were visually identified and subtracted from the data. During EEG pre-processing, two resting-state data sets and three event-related data sets (across 4 participants) were identified as having a large number of artifact contaminated epochs. These data sets contained <11 useable trials, below the recommended minimum of 20 trials (Cohen, 2014). Participants with an insufficient number of trials were excluded from further analysis, resulting in sample sizes of *N* = 21 for the resting-state analyses and *N* = 20 for the event-related analyses.

#### 2.7.2 Computing EEG connectivity as imaginary coherence (ImCoh)

tACS-induced changes in phase-based M1-M1 connectivity were assessed by analyzing the imaginary component of coherence (ImCoh; Nolte et al., 2004). Here, ImCoh indexed the consistency of phase angle differences (phase lag) between left M1 (tACS target) and right M1 signals. ImCoh was selected as the index of phase-based connectivity, as it has been shown to be less sensitive to volume conduction (Nolte et al., 2004). ImCoh values can range from 0 to 1, where 1 indicates perfect phase-synchronization (i.e., connectivity) between signals.

Using custom MATLAB scripts, ImCoh values were computed from the pre-processed resting-state data and the pre-processed event-related data. While electrodes C3 and C4 in international 10–20 EEG system correspond to the approximate position of the left M1 and right M1, respectively (Jurcak et al., 2007), we analyzed the activity of electrode clusters – a cluster of seven electrodes centered at C3 (C3 cluster: C3, FC3, C1, FC5, CP1, C5, CP3), and a cluster of seven electrodes centered at C4 (C4 cluster: CP2, CP4, C6, C2, FC6, FC4).

##### 2.7.2.1 Computing resting-state ImCoh

The time series of each electrode was convolved with complex Morlet wavelets for frequencies between 4 and 90 Hz, in 1 Hz increments (87 wavelet frequencies in total). The length of the wavelets started at 3 cycles for the lowest frequency, and logarithmically increased as the frequencies increased, such that the length was 13 cycles for the highest frequency. This approach provided a balance between temporal and frequency precision (Cohen, 2014). To minimize the effects of edge artifacts, analytic signals were only obtained from time windows of 400 to 1,600 ms (at 20 ms intervals) within each 2,000 ms epoch.

ImCoh values were computed for each electrode pair within the C3 and C4 clusters, using the following formula:


$$\left|\text{Imaginary}\;\left(\frac{S_{ij}\left(f\right)}{\sqrt{S_{ii}\left(f\right)S_{jj}\left(f\right)}}\right)\right|$$

Here, i and j represent the time series of each electrode. For frequency f, the cross-spectral density $S_{ij}\left(f\right)$ was taken from the complex conjugation of the complex Fourier transforms $x_{i}\left(f\right)$ and $x_{j}\left(f\right)$. Coherency was extracted by normalizing the cross-spectral density by the square root of the signals’ spectral power, $S_{ii}\left(f\right)$ and $S_{jj}\left(f\right)$. Then, the imaginary component of the resultant complex number was obtained. An estimate of M1-M1 ImCoh was obtained by averaging ImCoh values across all electrode pairs at each time point, frequency, and trial.

##### 2.7.2.2 Computing event-related ImCoh

**Figure 2:**
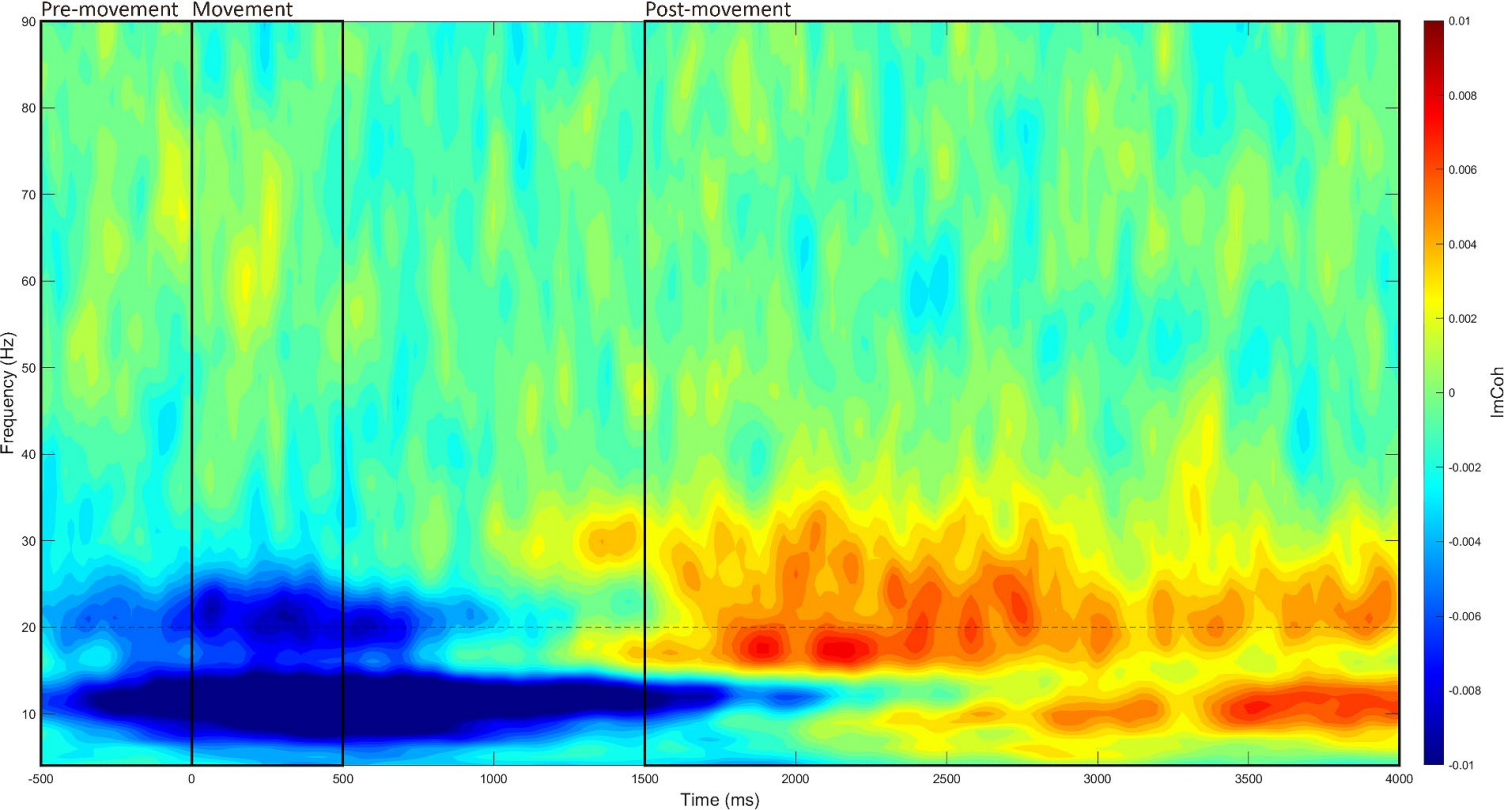
Grand-averaged event-related time-frequency M1-M1 ImCoh. Time-frequency ImCoh estimates were averaged across all recording blocks, participants, and stimulation intensities, then baselined to the period of –2,000 to –1,000 ms. Through visual inspection, the three movement periods were identified (marked in black boxes): (1) the pre-movement period (–500 to 0 ms), (2) the movement period (0 to 500 ms), and (3) the post-movement period (1,500 to 4,000 ms).

This process was identical to the resting state, with three exceptions. First, the time window of interest was –500 to 4,000 ms (at 20 ms intervals) relative to stimulus onset. Second, the ImCoh values were baselined to the period of –2,000 to –1,000 ms. Third, ImCoh estimates were separated into three different movement periods within each –500 to 4,000 ms window: (1) pre-movement period (–500 to 0 ms), (2) movement period (0 to 500 ms), and (3) post-movement period (1,500 to 4,000 ms). These periods were analyzed separately as each reflects a different aspect of movement, which are underpinned by slightly different mechanisms (Kilavik et al., 2013). The time window of each movement period was determined through visual inspection of the grand-averaged data (see Figure 2).

#### 2.7.3 Statistical analyses

In this study, the data were analyzed with mixed models instead of ANOVAs, due to mixed models accounting for inter-individual variability, as inter-individual response variability is a known issue in NIBS (for a review, see Guerra et al., 2020). Statistical analyses and visualization of the results were performed via customized scripts in MATLAB, and the software package R for Statistical Computing version 2023.09.0 + 463 (R Core Team, 2023), using packages ‘tidyverse’ (Wickham, 2023), ‘DescTools’ (Signorell, 2023), ‘janitor’ (Firke, 2023), ‘car’ (Fox et al., 2023), and ggplot2 (Wickham et al., 2023). The ‘lme4’ package (Bates et al., 2023) was used to contstruct mixed effects models, fitted by means of the lmer() function for linear mixed models (LMMs) or the glmer() function for generalized LMMs (GLMMs). Null hypothesis significance testing for main and interaction effects and post-hoc comparisons were performed with the ‘emmeans’ package (Lenth, 2023).

##### 2.7.3.1 Analyzing control measures

In addition to reporting the presence and strength of any tACS-related sensations, participants completed a questionnaire assessing their sleep quality and quantity (for the night before the session), as well as the amount of caffeine and alcohol consumed in the 12 h prior to each experimental session. Separate GLMMs were conducted to examine differences between stimulation intensities in the perceived strength of tACS sensations, as well as differences in pre-session sleep quality, sleep quantity, caffeine intake, and alcohol intake. GLMMs were selected over LMMs due to violations in normality. All models included participant-specific random intercepts. Model estimates were obtained for the fixed effect of INTENSITY (sham, 0.5 mA, 1.0 mA, and 1.5 mA). Akaike’s Information Criterion (AIC) values were used to determine the best distribution and function for each model (Burnham and Anderson, 2004).

##### 2.7.3.2 Analyzing changes in resting-state and event-related ImCoh

Trial-level estimates of resting-state ImCoh were obtained by averaging ImCoh values across the time points within each epoch. Trial-level estimates of event-related ImCoh were obtained by averaging ImCoh values across the time points within each movement period, within each epoch. Both resting-state and event-related ImCoh values were averaged across five frequency bands: theta (4–7 Hz), alpha (8–12 Hz), beta (13–30 Hz), and gamma (60–90 Hz).

The effects of tACS on resting-state ImCoh and event-related ImCoh were investigated with separate LMMs. Following a comparison of AIC values between LMMs and various GLMMs, LMMs were determined as having the best fit. For the event-related data, separate analyses were performed for each of the three movement periods (pre-movement, movement, post-movement). All models included participant-specific random intercepts to account for inter-individual variability in the data. Model estimates were obtained for the fixed effects of INTENSITY (sham, 0.5 mA, 1.0 mA, and 1.5 mA), TIME (pre, post_1_, and post_2_), and FREQUENCY (theta, alpha, beta, and gamma).

##### 2.7.3.3 Analyzing event-related peak beta

To explore the changes in endogenous-exogenous frequency differences within each movement period, we extracted each participants’ peak beta frequency for each trial, defined as the beta frequency with the greatest ERD/ERS in the corresponding time-frequency window. Trial-level estimates of endogenous-exogenous frequency differences were obtained by subtracting the stimulation frequency (20 Hz) from the peak beta frequency. The effect of tACS was investigated with separate LMMs for each movement period. All models included participant-specific random intercepts. Model estimates were obtained for the fixed effects of INTENSITY, TIME, and REGION. For all LMM analyses, statistical significance was set at α = 0.05, and significant effects were investigated with custom Bonferroni-corrected contrasts.

To explore the associations between endogenous-exogenous frequency differences and changes in event-related ImCoh, we performed cluster-based permutation tests using Spearman’s *ρ*. To overcome the multiple-comparisons problem, cluster-based permutation statistics are most appropriate for exploratory analyses in time-frequency data (Cohen, 2014). For each movement period, region, and stimulation intensity, we extracted participants’ trial-averaged: (1) pre-tACS exogenous-endogenous frequency difference; (2) percent-change in event-related ImCoh (Δ event-related ImCoh) between time points (i.e., between measurement blocks). Separate tests were conducted to examine associations with Δ event-related ImCoh between each time point (i.e., Δ pre to post_1_, Δ pre to post_2_, and Δ post_1_ to post_2_). For each test, sample points with values exceeding *α* = 0.05 were clustered according to spectral-temporal adjacency, with separate clusters for positive and negative values. The size of each cluster was determined by summing the absolute statistical values within it. The largest cluster size of each iteration was selected to form the permutation distribution. Clusters from the real data were compared to this permutation distribution, and cluster sizes that exceeded the 97.5^th^ percentile of this distribution were considered significant.

## 3 Results

Stimulation intensity-related changes in ImCoh over time were of primary interest in the present study. As such, all the main effects and only the highest level of interaction involving both INTENSITY and TIME as factors will be described in detail.

### 3.1 Control measures

Table 1 shows the descriptive statistics for each measure. GLMMs revealed that there were no significant differences between conditions in terms of sleep quality, sleep quantity, caffeine consumption, or alcohol consumption (*χ^2^*s ≤ 3.447, *p*s ≥ 0.328). In contrast, there was a significant difference in perceived sensations between stimulation intensities (*χ^2^* = 10.089, *p* = 0.018), with stronger sensations reported for higher stimulation intensities (see Table 1). *Post-hoc* comparisons revealed significantly stronger sensations reported for 1.5 mA stimulation relative to 0.5 mA stimulation (*z* = 3.018, *p* = 0.015, *d* = 0.954). There were no significant differences in sensations between the other stimulation intensities (|*z*s| ≤ 2.303, *p*s ≥ 0.128, |*d*s| ≤ 0.728). In light of this result, we tested whether the accuracy of participants guessing real vs. sham stimulation significantly differed between stimulation intensities. Accuracy (binary, i.e., correct vs. incorrect) data were analyzed with a GLMM using a binomial model with a probit link function. This model was determined as having the best fit, based on AIC values (Burnham and Anderson, 2004). Model estimates were obtained for the fixed effect of INTENSITY, revealing a significant main effect (*χ*^2^ (4, *N* = 21) = 8.931, *p* = 0.030). Custom Bonferroni corrected contrasts revealed a non-significant trend toward participants correctly guessing 1.0 mA and 1.5 mA stimulation as real, compared to participants correctly guessing sham (|*z*s| = 2.559, *p*s = 0.063, |*d*s| = 1.077). This was followed by an analysis of confidence ratings (scale: 1–10) with a LMM. Here, the main effect of INTENSITY was not significant (*χ*^2^ (4, *N* = 21) = 1.830, *p* = 0.608). Taken together, these results cast uncertainty over the efficacy of participant blinding to real and sham stimulation. However, research suggests that sham guessing may not affect the results. For example, Stanković et al. (2021) found that correct sham guessing did not moderate changes in memory performance post-transcranial direct current stimulation. Nonetheless, the subsequent results investigating the effect of tACS should be interpreted with caution.

**Table 1:** Descriptive statistics of control measures and strength of sensations perceived during tACS.

Control measure	Stimulation intensity			
	Sham	0.5 mA	1.0 mA	1.5 mA
Sleep quality (1–10)	6.77 ± 1.77	7.14 ± 1.80	6.30 ± 2.09	6.93 ± 1.36
Sleep quantity (hr)	6.66 ± 1.63	7.16 ± 1.84	6.61 ± 2.11	6.80 ± 1.15
Caffeine intake (mg)	58.23 ± 49.38	55.73 ± 49.67	55.73 ± 49.67	56.00 ± 57.23
Alcohol intake (unit)	0.00 ± 0.00	0.00 ± 0.00	0.09 ± 0.48	0.05 ± 0.21
Sensations during tACS	3.43 ± 2.89	2.35 ± 2.30	3.95 ± 3.20	4.50 ± 3.25

Data are expressed as mean (M) ± standard deviation (SD). Participants rated the intensity of their perceived sensations during tACS on a Likert scale ranging from 1 (nothing) to 5 (very strong).

### 3.2 Beta tACS induced intensity-dependent changes in resting-state ImCoh

The LMM analysis of resting-state ImCoh found significant main effects for all three factors: INTENSITY (sham, 0.5 mA, 1.0 mA, and 1.5 mA), TIME (pre, post_1_, and post_2_), and FREQUENCY (theta, alpha, beta, and gamma; all *χ^2^*s ≥ 84.93, all *p*s < 0.001). The analysis also revealed a two-way INTENSITY × TIME interaction (*χ^2^* (6, *N* = 21) = 195.50, *p* < 0.001). However, these interactions were mediated by a higher-order three-way interaction between INTENSITY, TIME, and FREQUENCY (*χ^2^* (18, *N* = 21) = 107.64, *p* < 0.001).

To facilitate interpretation of the significant three-way interaction, post-hoc analyses were conducted separately for each frequency band. Post-hoc analyses within each frequency band comprised three sets of comparisons. As the primary interest of the study was to examine the effect of stimulation intensity on resting-state ImCoh, the first set of comparisons aimed to determine which stimulation intensities showed significant ImCoh changes over time. To determine whether the changes were tACS-related, the second set of comparisons examined whether any of the real stimulation intensities showed significantly different changes in ImCoh compared to sham stimulation. If multiple real stimulation intensities showed significant differences from the sham, a third set of comparisons was performed on the real stimulation intensities to examine whether the change in ImCoh significantly differed between these stimulation intensities. It is important to note that there were significant differences in ImCoh between some of the stimulation intensities at baseline. Tables of these results can be found in the Supplementary Results. For this reason, comparisons between stimulation intensities were only performed on the relative change between two time points. It is possible that baseline differences may have affected the capacity for change, so comparisons between stimulation intensities should be interpreted with caution.

**Figure 3:**
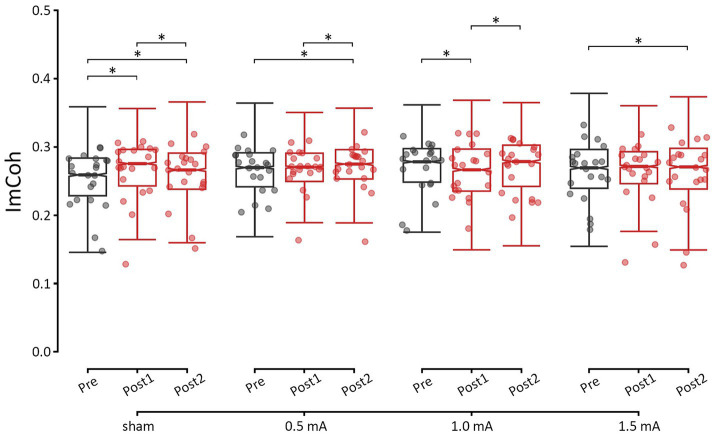
Changes in resting-state M1-M1 beta ImCoh for each time-point and stimulation intensity. *significant change between time-points at *α* = 0.05. Data points reflect participant averages. The height of the notches reflects the median +/− 1.57 x IQR/sqrt(n) where IQR is the interquartile range defined by the 25th and 75th percentiles and n is the number of data points.

Overall, changes in ImCoh varied between stimulation intensities in a frequency-specific manner. Relative to sham stimulation, none of the real stimulation intensities led to significant changes in theta and alpha ImCoh. In contrast, for the beta band, both 1.0 mA and 1.5 mA stimulation were followed by decreases in ImCoh, while sham and 0.5 mA stimulation led to increases in ImCoh. For gamma, all real stimulation intensities were followed by decreases in ImCoh. Here, we focus on describing the post-hoc analyses of resting-state ImCoh in the beta band, as this was the target frequency. Detailed results for each of the other frequency bands can be found in the Supplementary Results. As shown in Figure 3, increases in beta ImCoh were observed following sham and 0.5 mA stimulation, and decreases in beta ImCoh were observed following 1.0 mA and 1.5 mA stimulation. Immediately post-tACS (i.e., pre vs. post_1_), sham showed an increase in beta ImCoh (*z* = –7.425, *p* < 0.001, *d* = –0.262), while 1.0 mA stimulation showed a decrease (*z* = 2.728, *p* = 0.019, *d* = 0.095). By approximately 25 min post-tACS (i.e., pre vs. post_2_), both sham and 0.5 mA stimulation showed an increase in beta ImCoh (*|z*s| ≥ 2.580, *p*s ≤ 0.030, |*d*s| ≥ 0.092), and 1.5 mA showed a significant decrease (*z* = 3.889, *p* < 0.001, *d* = 0.137). Between post_1_ and post_2_, sham and 1.0 mA ImCoh began to shift back toward baseline (*|z*s| ≥ 2.672, *p*s ≤ 0.023, |*d*s| ≥ 0.093), though 0.5 mA ImCoh significantly increased (*z* = −2.832, *p* = 0.014, *d* = −0.100) and 1.5 mA ImCoh remained suppressed (*z* = 2.326, *p* = 0.060, *d* = 0.082). Taken together, these results indicate that 0.5 mA tACS induced a delayed increase in phase-based connectivity between left and right M1. Further, 1.0 mA tACS immediately suppressed connectivity, while 1.5 mA tACS induced a delayed suppression.

### 3.3 Beta tACS induced little change in event-related ImCoh

Overall, results indicated that, for any movement period, tACS had a limited impact on the event-related functional connectivity between M1s. The most notable change was observed in the movement period, where there was a significant decrease in broadband ImCoh (4–90 Hz) following 1.5 mA stimulation. Importantly, there were no significant differences in ImCoh between any of the stimulation intensities at baseline. Tables of these results can be found in the Supplementary Results. The following sections provide a detailed description of the findings for each movement period.

#### 3.3.1 Pre-movement ImCoh (–500–0 ms)

The LMM analysis of pre-movement ImCoh found a significant main effect for FREQUENCY (*χ^2^* = 147.588, *p* < 0.001), but not INTENSITY or TIME (*χ^2^*s ≤ 4.845, *p*s ≥ 0.183). The analysis also revealed a significant two-way INTENSITY × TIME interaction (*χ^2^* = 13.428, *p* = 0.037). However, the three-way interaction of INTENSITY, TIME, and FREQUENCY was not significant (*χ^2^* = 15.696, *p*s ≥ 0.613). These results indicate that tACS induced stimulation intensity-specific changes in pre-movement ImCoh. However, these changes were not frequency-specific, indicating that beta tACS did not selectively modulate pre-movement beta ImCoh. The highest level of interaction – the two-way INTENSITY × TIME interaction – was further investigated with post-hoc comparisons.

**Figure 4:**
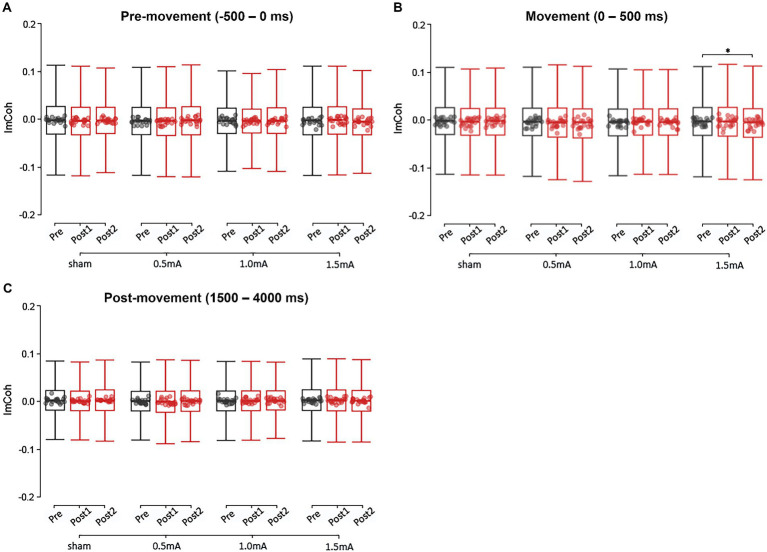
Event-related changes in broadband ImCoh (4–90 Hz), at each current-intensity, for the pre-movement **(A)**, movement **(B)**, and post-movement **(C)** periods. ImCoh values have been baseline-normalised to the period of –2,000 to –1,000 ms. *significant change between time-points at *α* = 0.05. Data points reflect participant averages. The height of the notches reflects the median +/− 1.57 x IQR/sqrt(n) where IQR is the interquartile range defined by the 25th and 75th percentiles and n is the number of data points.

Figure 4A shows the pre-movement ImCoh values for each time point and stimulation intensity. There were no significant differences in pre-movement ImCoh between stimulation intensities at baseline (|*z*s| ≤ 2.068, *p*s ≥ 0.232, |*d*s| ≤ 0.050). Post-hoc analyses revealed that none of the stimulation intensities showed a significant change in ImCoh between any of the time points (|*z*s| ≤ 2.273, *p*s ≥ 0.069, |*d*s| ≤ 0.056). The only significant difference was between 0.5 mA and 1.5 mA stimulation from post_1_ to post_2_: 0.5 mA stimulation showed an increase in ImCoh while 1.5 mA stimulation showed a decrease (*z* = 3.254, *p* = 0.021, *d* = 1.726). However, the changes following 0.5 mA and 1.5 mA did not significantly differ from sham (|*z*s| ≤ 3.254, *p*s ≥ 0.273, |*d*s| ≤ 1.726), indicating that these stimulation intensities did not induce a marked change in ImCoh.

#### 3.3.2 Movement ImCoh (0–500 ms)

The LMM analysis of movement ImCoh found a significant main effect for INTENSITY and FREQUENCY (*χ^2^*s ≥ 18.939, *p*s < 0.001), but not TIME (*χ^2^* = 5.212, *p* = 0.074). The analysis also revealed a significant two-way INTENSITY × TIME interaction (*χ^2^* = 13.854, *p* = 0.031). However, the three-way interaction of INTENSITY, TIME, and FREQUENCY was not significant (*χ^2^* = 14.364, *p*s ≥ 0.705). These results indicate that tACS induced stimulation intensity-specific changes in movement ImCoh, but not frequency-specific changes. The highest level of interaction – the two-way INTENSITY × TIME interaction – was further investigated with post-hoc comparisons.

Figure 4B shows the movement ImCoh values for each time-point and stimulation intensity. There were no significant differences in movement ImCoh between stimulation intensities at baseline (|*z*s| ≤ 2.268, *p*s ≥ 0.140, |*d*s| ≤ 0.055). Post-hoc analyses revealed that there was a significant decrease in movement ImCoh from pre to post_2_ following 1.5 mA stimulation (*z* = 2.557, *p* = 0.032, *d* = 0.064), indicating that 1.5 mA beta tACS suppressed phase-based connectivity between left and right M1 during movement execution. There were no other changes in movement ImCoh (|*z*s| ≤ 2.279, *p*s ≥ 0.068, |*d*s| ≤ 0.055).

#### 3.3.3 Post-movement ImCoh (1,500–4,000 ms)

The LMM analysis of post-movement ImCoh found significant main effects for INTENSITY and FREQUENCY (*χ^2^*s ≥ 20.550, *p*s < 0.001), but not TIME (*χ^2^* = 1.580, *p* = 0.454). However, neither the two-way INTENSITY × TIME interaction was significant (*χ^2^* = 7.278, *p* = 0.296), nor the three-way INTENSITY × TIME × FREQUENCY interaction (*χ^2^* = 10.782, *p*s ≥ 0.904). These results indicate that real tACS did not induce stimulation intensity-or frequency-specific changes in post-movement ImCoh. Figure 4C shows the post-movement ImCoh values for each time-point and stimulation intensity.

### 3.4 Beta tACS did not cause a shift in event-related endogenous-exogenous frequency differences

We also examined each movement period to determine whether any of the stimulation intensities caused a shift in endogenous-exogenous frequency differences (i.e., differences between the participants’ peak beta frequency for ImCoh and the tACS frequency of 20 Hz). The LMM analyses revealed a significant main effect for INTENSITY in the movement and post-movement periods (*χ^2^*s ≥ 7.983, *p*s ≤ 0.046). All other main effects were non-significant (*χ^2^*s ≤ 2.456, *p*s ≥ 0.293). The two-way INTENSITY × TIME interaction was not significant any movement period (*χ^2^*s ≤ 6.078, *p*s ≥ 0.415), indicating that none of the tACS conditions shifted the ImCoh peak beta frequency within any of the three movement periods.

### 3.5 Pre-tACS endogenous-exogenous frequency differences were associated with post-tACS changes in event-related ImCoh

**Figure 5:**
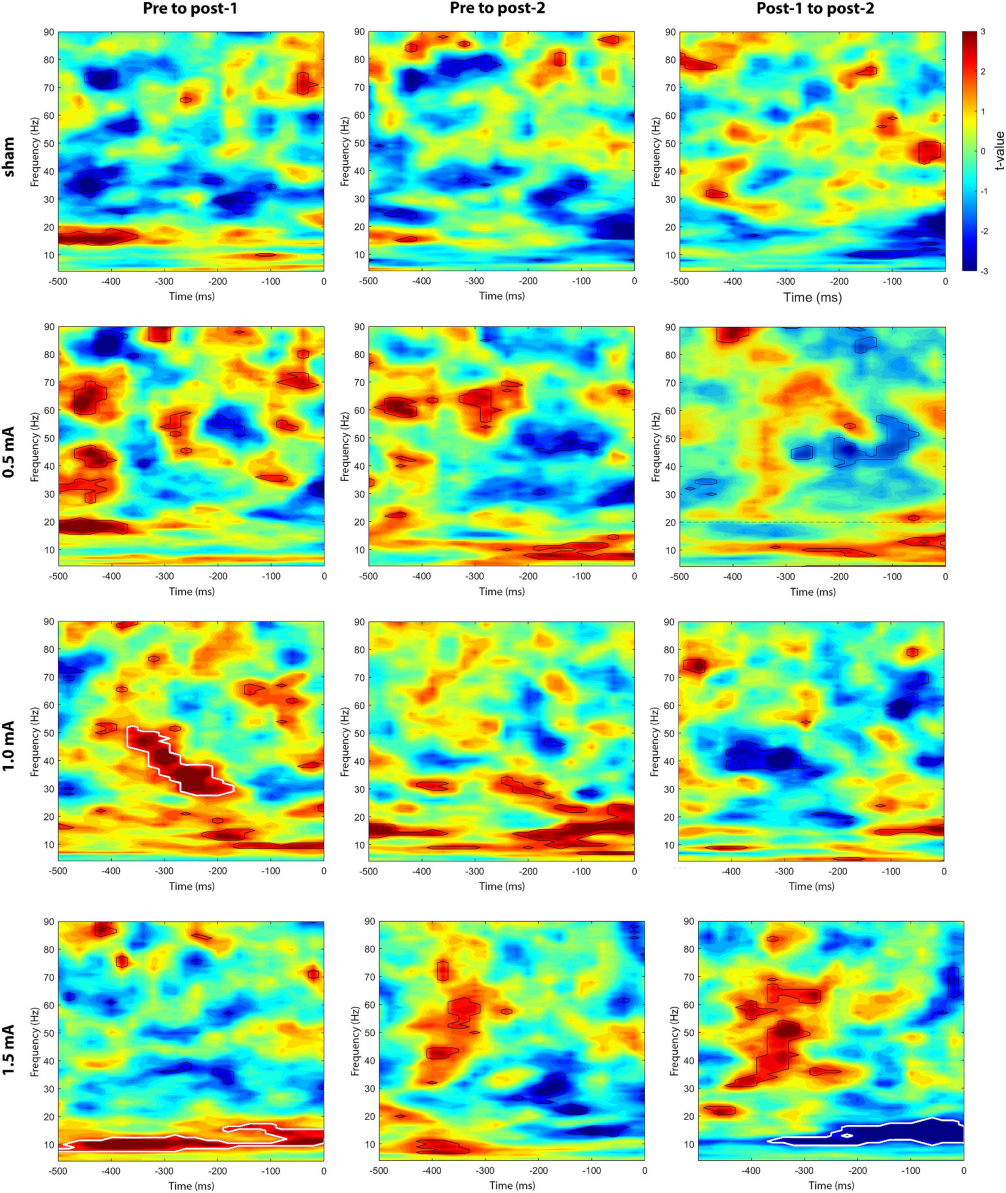
Cluster-based correlations (Spearman’s *ρ*) between endogenous-exogenous frequency differences and changes in event-related M1-M1 ImCoh, for the pre-movement period. Areas bordered with thick white lines indicate clusters with significant correlations.

**Figure 6:**
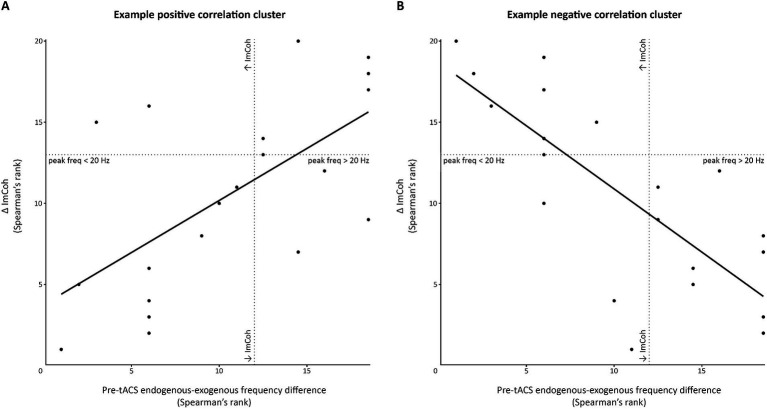
Scatterplot demonstrating significant correlation clusters observed in the pre-movement period. This figure demonstrates the significant clusters of positive **(A)** and negative **(B)** correlations between the pre-tACS endogenous-exogenous frequency difference and the change in ImCoh post-tACS (Δ ImCoh). In this example, data were extracted from the pre-movement period, following 1.5 mA stimulation, at time-frequency points within the significant clusters. For **(A)**, data have been extracted from the pre to post1 change at –380 ms and 10 Hz. For **(B)**, data have been extracted from the post_1_ to post_2_ change at –100 ms and 15 Hz. Dotted lines indicate points where Δ ImCoh and/or the endogenous-exogenous frequency difference equals 0.

We also explored whether the endogenous-exogenous frequency differences were associated with the change in ImCoh (ΔImCoh) following tACS. We performed separate cluster-based correlations on each movement period, time-point comparison, and stimulation intensity. Significant cluster-corrected correlations were only observed for the pre-movement period, following 1.0 mA and 1.5 mA stimulation. From pre to post_1_, there were significant positive correlation clusters following both 1.0 mA and 1.5 mA stimulation (see Figure 5). Following 1.0 mA stimulation, the significant positive correlation cluster was between 28–52 Hz and between –360 – –180 ms. Following 1.5 mA stimulation, the significant positive correlation cluster was between 8–17 Hz and between –500 – 0 ms. To better understand the nature of the correlations, we generated a scatterplot of endogenous-exogenous frequency differences and ΔImCoh values at several time-frequency points within the significant clusters (see Figure 6A for an example point). Relative to an endogenous-exogenous frequency difference of 0 Hz, the results were bidirectional, suggesting that individuals with peak frequencies further below 20 Hz showed greater decreases in ImCoh following 1.0 mA and 1.5 mA tACS, and individuals with peak frequencies further above 20 Hz showed greater increases in ImCoh following 1.0 mA and 1.5 mA tACS.

From pre to post_2_, there were no significant correlation clusters following any stimulation intensity. From post_1_ to post_2_, a significant correlation cluster was only observed following 1.5 mA stimulation. This cluster was negative and located between 10–19 Hz and between –360– –180 ms. Again, the results were bidirectional (see Figure 6B), suggesting that individuals with peak frequencies further below 20 Hz showed greater increases in ImCoh following 1.5 mA tACS, and individuals with peak frequencies further above 20 Hz showed greater decreases in ImCoh. It is likely that the change from positive to negative correlation between post_1_ and post_2_ reflects a return to baseline ImCoh levels in individuals who showed changes in ImCoh from pre to post_1_.

## 4 Discussion

In the present study, we investigated the effect of different beta tACS intensities on both resting-state and event-related M1-M1 functional connectivity. For resting-state connectivity, we observed intensity-dependent changes in beta ImCoh: sham and 0.5 mA stimulation increased beta ImCoh, while 1.0 mA and 1.5 mA stimulation decreased beta ImCoh. Importantly, changes in resting-state ImCoh were frequency-specific: relative to sham stimulation, significant changes were observed in the beta and gamma bands, but not theta or alpha bands. For event-related connectivity, 1.5 mA stimulation decreased broadband ImCoh (4–90 Hz) during movement. Notably, the changes in pre-movement ImCoh following 1.0 mA and 1.5 mA stimulation were significantly associated with participants’ pre-tACS endogenous-exogenous frequency difference.

### 4.1 Effect of tACS on resting-state ImCoh

#### 4.1.1 Beta ImCoh increased immediately following sham but not real stimulation

Sham stimulation was expected to induce the sensations typically perceived during tACS but was not expected to induce a lasting neurophysiological effect (Gandiga et al., 2006; Ambrus et al., 2012; Woods et al., 2016). However, following sham stimulation, there was a significant increase in beta ImCoh at post_1_ (~5 min post-tACS), which remained elevated at post_2_ (~25 min post-tACS). This increase in M1-M1 connectivity might be explained by the ≥25 min period of reduced motor input from pre to post_1_. Indeed, Todd et al. (2006) found that 20 min of hand inactivity increased corticospinal excitability. While the relationship between corticospinal excitability and M1-M1 beta connectivity remains unknown, research indicates that corticospinal excitability is greater during periods of high M1-M1 connectivity within the mu band (8–13 Hz; Vetter et al., 2023). Similar to the beta band, mu activity has been implicated in movement, showing comparable patterns of ERD/S (Pfurtscheller and Neuper, 1994; Pfurtscheller and Lopes Da Silva, 1999). In the current study, it is plausible that the observed increase in beta ImCoh following sham reflects an increase in corticospinal excitability due to hand inactivity, though this is speculative and requires further investigation. Alternatively, the increase in ImCoh might have resulted from spontaneous fluctuations in connectivity (Chang and Glover, 2010) or variability between EEG measures (Höller et al., 2017), though sensorimotor beta ImCoh has been shown to have good-to-excellent test–retest reliability (Popov et al., 2023). It is important to note that, in contrast to sham, the real stimulation intensities did not induce increases in beta ImCoh at post_1_, indicating that real tACS might have interacted with beta oscillations to prevent the increase in ImCoh observed following sham stimulation.

#### 4.1.2 The frequently used tACS intensity of 1.0 mA decreased resting-state M1-M1 beta ImCoh

We found that 1.0 mA tACS induced a significant decrease in beta ImCoh from pre to post_1_, which was distinct from the response following sham stimulation (i.e., an increase in ImCoh), indicating that this decrease was likely induced by tACS. Notably, the effect was short lasting, beginning to return to baseline levels at post_2_. Only two other studies have examined the effect of left M1 beta tACS on motor network functional connectivity. Weinrich et al. (2017) did not observe significant changes in M1-M1 BOLD connectivity following 20 Hz tACS at 1.0 mA to left M1. However, they found that the positive relationship between M1-M1 connectivity and overall motor network strength (observed during 5 Hz and sham stimulation) disappeared during 20 Hz tACS. This indicates that 1.0 mA beta tACS might reduce coupling between stimulated left M1 and the unstimulated regions within the motor network. This notion of reduced coupling might explain the decrease in beta ImCoh that was observed in the present study. In contrast to our findings, Wischnewski et al. (2019) did not observe a significant change in M1-M1 phase-based EEG connectivity after applying 15 min of 1.0 mA beta tACS to left M1. Aftereffects following 1.0 mA beta tACS have also been inconsistent for M/EEG power, with studies reporting increases (Nakazono et al., 2020; Suzuki et al., 2022) and others reporting no change (Wach et al., 2013; Rumpf et al., 2019; Lafleur et al., 2021). Additionally, mixed findings have been reported for corticospinal excitability and motor function (for a review see Rostami et al., 2024). For example, Pozdniakov et al. (2021) reported no change in corticospinal excitability following 15 min of beta tACS to left M1. Inconsistent results might be explained by differences in stimulation parameters (i.e., electrode montage and stimulation duration) or the approach/sensitivity to statistical analysis (i.e., analysis of variance vs. LMM). In the current study, LMMs with participant-specific random intercepts were used to partially account for inter-individual variability.

Taken together, available findings indicate that 1.0 mA of unifocal beta tACS might modulate the functional connectivity of the motor network, by reducing coupling between stimulated and unstimulated regions, though further investigation is warranted to confirm these effects. Importantly, the communication through coherence theory (Fries, 2005, 2015) suggests that reduced coupling between brain regions will be less efficient, affecting the processes tied to these regions. It will be important for future studies to examine the functional consequences of unifocal tACS on both unilateral and bilateral movement.

#### 4.1.3 Changes in resting-state M1-M1 beta ImCoh were intensity-dependent and non-linear

We hypothesized that beta tACS would linearly decrease resting-state beta ImCoh. This hypothesis was not supported by the results, as the stimulation intensity modulated ImCoh in a non-linear manner. At post_1_, a significant decrease in beta ImCoh was observed following 1.0 mA stimulation, while no changes were observed following 0.5 mA or 1.5 mA stimulation. At post_2_, a delayed increase was observed following 0.5 mA stimulation, and a delayed decrease was observed following 1.5 mA stimulation, though this decrease was not different to 1.0 mA stimulation.

As previously mentioned, real stimulation at all intensities tested here might have interacted with beta oscillations to prevent an increase in ImCoh at post_1_ (observed following sham). The delayed increase in ImCoh following 0.5 mA stimulation could suggest a diminishing impact at this intensity, indicating that the 0.5 mA stimulation may not have elicited as pronounced or enduring an effect in preventing an increase in ImCoh, relative to the higher stimulation intensities. The decrease in ImCoh following both 1.0 mA and 1.5 mA stimulation might reflect the reduced coupling between M1s. However, it is unclear why the timeline of the tACS-induced change in ImCoh for these two stimulation intensities differed: the decrease following 1.0 mA stimulation was observed at post_1_ and began to return to baseline levels by post_2_, while the response to 1.5 mA stimulation was delayed, potentially reflecting late plasticity-like mechanisms. Indeed, there have been reports of late plasticity-like changes in corticospinal excitability following 20 Hz and 250 Hz tACS, when applied at 1.0 mA (Moliadze et al., 2010; Wischnewski et al., 2019). It is also unclear why we observed a plateau in stimulation efficacy – relative to 1.0 mA stimulation, 1.5 mA stimulation did not induce a significantly greater change in ImCoh. Further, it is unclear why the intensity-response relationship observed in the current study was non-linear. In human studies, tACS has shown both linear (Moliadze et al., 2012) and non-linear responses (De Koninck et al., 2021; Shorafa et al., 2021; Wang et al., 2022). These non-linear effects might be due to homeostatic mechanisms acting to regulate appropriate neural spike timing (Shorafa et al., 2021), highlighting the complex nature of the intensity-response relationship.

#### 4.1.4 Changes in resting-state ImCoh were frequency-specific

In addition to the changes in beta ImCoh, we examined the effect of beta tACS on multiple oscillatory frequency bands to understand its broader impact on functional connectivity. Relative to sham, there was no change in resting-state theta and alpha ImCoh. However, significant decreases in gamma ImCoh were observed following all real stimulation intensities. The significant changes in gamma might be the result of cross-frequency coupling, where two (or more) oscillatory frequencies are paired in phase and/or amplitude and exert influence on one another (Jensen and Colgin, 2007; Canolty and Knight, 2010; Hyafil et al., 2015). Several studies have reported cross-frequency changes in oscillations following tACS of other frequencies (Helfrich et al., 2016; Jone et al., 2020; e.g., de la Salle et al., 2023). However, it is unclear why, in the current study, only resting-state gamma oscillations were modulated by beta tACS (compared to theta and alpha). The modulation of gamma might be explained by the functional interaction between beta and gamma within the motor network: beta-gamma coupling has been suggested to play an important role in motor control (De Hemptinne et al., 2013; Gong et al., 2022). Alternatively, this result might reflect a feedback loop, where beta tACS altered the balance of excitatory and inhibitory circuits, leading to changes in gamma oscillations (Michalareas et al., 2016). Taken together, these findings demonstrate that beta tACS can induce cross-frequency modulation of M1-M1 connectivity.

### 4.2 Effect of tACS on event-related ImCoh

#### 4.2.1 The standard tACS intensity of 1.0 mA did not modulate event-related M1-M1 ImCoh

In contrast to resting-state ImCoh, 1.0 mA stimulation did not significantly modulate the event-related changes in M1-M1 beta ImCoh during any movement period. A speculative explanation for the difference between resting-state and event-related results might be that the beta connectivity patterns crucial for movement preparation, execution, and termination superseded the observed resting-state changes induced by 1.0 mA tACS. Alternatively, 1.0 mA stimulation may have been insufficient to modulate the mechanisms underlying the event-related connectivity patterns between M1s.

Though we did not observe pre/post changes in event-related connectivity, it is possible that the stimulation might have induced changes during stimulation that were not captured in the present study. Research indicates that, relative to pre/post assessments, assessment of the tACS response during stimulation captures greater changes in motor neurophysiology (Pozdniakov et al., 2021) and behavior (Hu et al., 2022). Unfortunately, the current study was limited to examining tACS aftereffects, due to the technical challenges associated with stimulation artifacts contaminating EEG recordings (Noury et al., 2016; Neuling et al., 2017; Noury and Siegel, 2018; Asamoah et al., 2019; Kasten and Herrmann, 2019). Future studies might overcome this limitation with devices that support concurrent stimulation and recording, as well as developments in artifact removal techniques (Fehér and Morishima, 2016).

#### 4.2.2 Evidence of intensity-dependent changes in event-related M1-M1 ImCoh

We hypothesized that the changes in event-related M1-M1 beta ImCoh would be intensity-dependent. However, the interaction between INTENSITY, TIME, and FREQUENCY was not statistically significant, suggesting that none of the real stimulation intensities modulated ImCoh in a frequency-specific manner. Instead, we observed intensity-related changes in broadband ImCoh (4–90 Hz). For the pre-movement period, the effects of 0.5 mA and 1.5 mA stimulation significantly differed: from post_1_ to post_2_, 0.5 mA stimulation led to an increase in broadband ImCoh, while 1.5 mA stimulation led to a decrease. However, neither of these real stimulation intensities showed a significant change in pre-movement ImCoh relative to sham, indicating that the differences might have been due to spontaneous fluctuations in ImCoh. For the movement period, the only significant change was a delayed decrease in ImCoh following 1.5 mA stimulation. This event-related result aligns with the delayed decrease in resting-state beta ImCoh following 1.5 mA stimulation, which might reflect late plasticity-like mechanisms which are activated in an intensity-dependent manner. In contrast, for the post-movement period, there were no significant changes in ImCoh. The different responses across movement periods might be explained by the different neural processes underpinning each movement period (Kilavik et al., 2013), as differences in brain states have been shown to affect the tACS response (e.g., Shorafa et al., 2021; Wang et al., 2022).

### 4.3 Beta tACS did not modulate event-related peak beta frequencies

If beta tACS modulated oscillations, it is plausible that the stimulation might have shifted peak beta frequencies closer toward the exogenous frequency. We explored this by examining changes in the endogenous-exogenous frequency difference from pre to post-tACS. For all movement periods, the endogenous-exogenous frequency difference did not change following tACS of any intensity, indicating that beta tACS did not induce a lasting change in participants’ endogenous peak beta frequency. This is unsurprising, given the lack of beta-specific aftereffects in event-related ImCoh.

### 4.4 Changes in event-related ImCoh were associated with pre-tACS endogenous-exogenous frequency differences

We also explored whether participants’ endogenous-exogenous frequency difference was associated with their event-related ImCoh response. Evidence indicates that personalizing stimulation to participants’ peak frequency can induce greater neuromodulation relative to both sham and fixed frequency tACS (e.g., Ayanampudi et al., 2023). If personalized frequencies induce greater neuromodulation, then individual pre-tACS endogenous-exogenous frequency difference in the current study might have been associated with the post-tACS change in event-related ImCoh. For the pre-movement period, from pre to post_1_, the endogenous-exogenous frequency difference was positively correlated with the change in ImCoh following both 1.0 mA and 1.5 mA tACS. This finding aligns with the results observed by Kudo et al. (2022), in which an association between pre-tACS endogenous-exogenous frequency differences and the response to 20 Hz oscillatory transcranial direct current stimulation (otDCS): endogenous-exogenous frequency differences were negatively associated with changes in corticomuscular coherence. Interestingly, in the current study, the frequencies of the positive clusters differed between these two stimulation intensities. Following 1.0 mA stimulation, participants with pre-movement peak frequencies further below 20 Hz tended to show greater decreases in M1-M1 ImCoh between 28 and 52 Hz. In contrast, following 1.5 mA stimulation, participants with pre-movement peak frequencies further below 20 Hz tended to show greater decreases in M1-M1 ImCoh between 8 and 17 Hz. This indicates that, in movement preparation, the alignment between the stimulation frequency and participants’ peak beta frequencies might have affected the extent of reduced M1-M1 coupling from: (1) high-beta/low-gamma activity following 1.0 mA stimulation, and (2) the extent of reduced M1-M1 coupling from alpha/mu and low-beta activity following 1.5 mA stimulation. It is unclear why the frequencies of the significant clusters between the two stimulation intensities differed. It is also unclear why the relationship was only significant for the pre-movement period and these two stimulation intensities. Taken together, it appears that there may be a complex interaction between the stimulation intensity and frequency of left M1 beta tACS to effectively modulate the connectivity between M1s. It will be important for future studies to investigate whether personalizing 1.0 mA and 1.5 mA stimulation to the individual beta frequency induces a greater change in M1-M1 connectivity during movement preparation.

### 4.5 Limitations

Changes in phase-based M1-M1 connectivity were assessed via ImCoh. ImCoh is one of several phase-based connectivity methods that can be used to examine connectivity; these methods differ in their approach of examining signal interactions and thus offer somewhat different insights into connectivity (Cohen, 2014). For the current study, ImCoh was selected due to its advantage in mitigating issues of volume conduction when analysing connectivity at the sensor level (Nolte et al., 2004), as well as its reported robustness (Sanchez Bornot et al., 2018). Nonetheless, it is important to note that ImCoh can only detect connectivity in time-lagged signals (Nolte et al., 2004), and that the tACS-induced changes in ImCoh might not necessarily be reflected in other connectivity metrics (Wang et al., 2014).

It is also important to acknowledge that this study was limited to examining the offline (i.e., post-stimulation) effect of tACS on M1-M1 connectivity. During stimulation, the tACS electrodes obstructed the EEG electrodes that recorded activity from the target site, and the stimulation artifact would have contaminated activity recorded from nearby electrodes (Asamoah et al., 2019; Kasten and Herrmann, 2019; Neuling et al., 2017; Noury et al., 2016; Noury and Siegel, 2018). Previously, studies have reported differences between online and offline assessment in the modulation of neurophysiology (e.g., corticospinal excitability) in (e.g., corticospinal excitability in Feurra et al., 2011 and Pozdniakov et al., 2021) and motor function (e.g., bimanual coordination in Heise et al., 2019). Differences have also been observed across studies comparing tACS-induced changes in online and offline M/EEG. For example, greater changes in parieto-occipital alpha power and gamma coherence have been reported during online assessment (Helfrich et al., 2014a, 2014b). As no studies have directly assessed the online and offline effects of beta tACS on M1-M1 connectivity, it would be valuable for future research to investigate this with devices that support concurrent stimulation and recording, as well as sophisticated artifact removal techniques.

Additionally, the current study only used one frequency, i.e., 20 Hz, in examining the effect of tACS on M1-M1 connectivity. Consequently, it is unclear whether the intensity-dependent changes in connectivity were specific to beta stimulation. Considering that M1 tACS has shown frequency-dependent changes in corticospinal excitability and motor function (for a review, see Rostami et al., 2024), future studies should investigate whether the frequency-dependent effects of M1 tACS extend to changes in M1-M1 connectivity. Specifically, future studies should compare changes in connectivity across a broad spectrum of stimulation frequencies, including other frequencies that have been strongly implicated in the motor system (e.g., alpha and gamma; Pfurtscheller and Lopes Da Silva, 1999). Furthermore, it is unclear whether the effects were specific to the HD montage, or whether similar effects would be observed following stimulation with the conventional bipolar montage. Indeed, the two montages differ in electric field spread (Datta et al., 2008; Dmochowski et al., 2011), and differences in electric field spread have been associated with differences in neuromodulation (for a review, see Hunold et al., 2023). For example, Kasten et al. (2019) found that inter-individual variability in the tACS-induced modulation of alpha power is predicted by the modelled electric field spread. It would be valuable for future research to examine whether the connectivity changes reported in the current study would be observed when tACS is applied with the conventional bipolar montage.

Last, the results of the current study were impacted by both intra-and inter-individual variability. Notably, between-session intra-individual variability largely contributed to the significant baseline differences in resting-state ImCoh. Previous studies have reported low to moderate test–retest reliability for ImCoh (Colclough et al., 2016; Candelaria-Cook and Stephen, 2020), which likely results from spontaneous between-session fluctuations in neural oscillations and noise (for a detailed discussion regarding the variability of resting-state connectivity metrics, see Colclough et al., 2016). Importantly, the GLMM accounted for inter-session variability by including participant-specific random intercepts. This allows each participant to have their own baseline level, thus modeling the individual differences appropriately. Further, participant (intercept) variance was low (variance = 0.014, SD = 0.120), and a caterpillar plot showed that the random intercepts were centered around zero and within a reasonable range. As such, we do not believe that the intra-individual variability significantly biased our interpretation of the tACS-induced changes. Furthermore, results of the current study show large inter-individual differences in tACS response. While intensity-dependent changes in ImCoh were observed at the group level, there were inter-individual differences in the response to tACS. For example, at post_1_, ~28% of participants showed a decrease in resting-state beta ImCoh at left M1 following 1.0 mA stimulation, while ~10% showed an increase and ~ 62% showed no change (defined as <10% change). Inter-individual response variability is a prevalent issue within the tACS literature, which limits the potential of the technique. Various factors may contribute to inter-individual response variability, including differences in anatomical, neurochemical, and demographic characteristics (for a review of these factors, see Vergallito et al., 2022). Future research should strive to improve the efficacy of tACS at the individual level through the personalization of parameters, such as the stimulation frequency (for a recent review of stimulation personalization, see Wansbrough et al., 2024).

## 5 Conclusion

The current study investigated the effect of left M1 beta tACS on resting-state and event-related M1-M1 connectivity. Our results suggest that unifocal beta tACS decreases connectivity between the stimulated left M1 and unstimulated right M1. Additionally, we demonstrated that the tACS intensity impacted the modulation of connectivity in non-linear manner. To our knowledge, this is the first study that has systematically explored how the stimulation intensity affected the network response to tACS. Finally, we showed that the importance of considering the individual peak frequency when evaluating the tACS response. This knowledge has important implications for understanding the effects that beta tACS might have on motor function and can be used toward optimizing tACS protocols for research and clinical settings.

## Data Availability

The raw data supporting the conclusions of this article will be made available by the authors, without undue reservation.
