# Supplementary material for: Beta tACS of varying intensities differentially affect resting-state and movement-related M1-M1 connectivity
Source: Front Neurosci. 2024 Sep 20;18:1425527. doi: 10.3389/fnins.2024.1425527 (PMC11450697; doi:10.3389/fnins.2024.1425527)
Supplement: Supplementary file 1 [file Data_Sheet_1.pdf]

Supplementary Material

1 Supplementary Methods

1.1 Determining the hand area of M1 for tACS

To determine the sites for tACS electrode placement, surface electromyographic (EMG) recordings and single-pulse TMS were used to identify the left M1 representation of the FDI. EMG activity was recorded from the right FDI using Ag/AgCl cup electrodes in a belly-tendon configuration. The raw electrical signal from the target muscle was amplified 1000 times (CED 1902 amplifier) and band pass filtered (20-1000 Hz). The signal was then digitized at a sampling value of 5000Hz (CED Power1401).

TMS was delivered with Magstim 200^2 (Magstim, Whitland, Dyfed, UK) via a 90 mm figure-of-eight coil. The coil was placed tangentially to the scalp with the handle pointed backward, 45° away from the midline (that is, at the optimal coil orientation for inducing posterior-anterior current flow in M1; Janssen et al., 2015). The optimal site on the scalp for eliciting motor evoked potentials (MEPs) in the FDI was located using single pulses of TMS. The optimal site was defined as the site that evoked the largest and most reliable MEPs (Rossini et al., 1994, 2015). Once the optimal site was determined, the scalp was marked for placement of the center tACS electrode.

1.2 Electric field models

Electric field simulations of the 4×1 HD-tACS montage were conducted with SimNIBS (v.3.2.0; Thielscher et al., 2015) using finite-element methods. The SimNIBS "headreco" function was used to generate a head model from the MNI152 template brain. For all simulations, electrodes were modeled as rubber discs (conductivity: 29.400 S/m) that were 20 mm in diameter and 2 mm thick, with conductive gel (conductivity: 1.000 S/m) of 2 mm thickness underneath. The center electrode was placed over the C3 coordinate of the 10-20 system, and the four return electrodes were each placed at a radius of 50 mm. Supplementary Table 1 shows the mean electric field strength at left M1, as well as the current focality, for each stimulation intensity. The mean electric field strength at left M1 was computed by means of binary masks, based on the human motor area template (Archer et al., 2018).

Supplementary Table 1. Simulated electric field strength and focality

Stimulation Intensity	Mean Electric Field Strength at Left M1	Electric Field Focality	
		Vol_{75}	Vol_{50}
0.5mA	2.80E-03 V/m	7.78E+03	2.47E+03
1.0mA	5.65E-03 V/m	7.78E+03	2.47E+03
1.5mA	8.46E-03 V/m	7.78E+03	2.47E+03

2 Supplementary Results

2.1 Baseline differences in resting-state ImCoh between stimulation intensities

In the main manuscript, an LMM analysis was performed to examine the effect of tACS on resting-state ImCoh. Model estimates were obtained for the fixed effects of INTENSITY (sham, 0.5 mA, 1.0 mA, and 1.5 mA), TIME (pre, $post_1$, and $post_2$), and FREQUENCY (theta, alpha, beta, and gamma). Results revealed significant differences in ImCoh between some of the stimulation intensities at baseline. As the highest level of significant interaction was a three-way interaction between INTENSITY, TIME, and FREQUENCY, the baseline differences for each frequency band have been reported in Supplementary Table 2.

Supplementary Table 2. Baseline differences in ImCoh between stimulation intensities

**Frequency band**	**Intensity comparisons**	***z***	***p***	**Effect size (*d*)**
Theta	1.5mA - 1.0mA	-3.566	0.002**	-0.125
	1.5mA - 0.5mA	-0.177	1.000	-0.006
	1.5mA - sham	5.946	< 0.001***	0.211
	1.0mA - 0.5mA	3.376	0.004**	0.119
	1.0mA - sham	9.491	< 0.001***	0.337
	0.5mA - sham	6.101	< 0.001***	0.218
Alpha	1.5mA - 1.0mA	-0.159	1.000	-0.006
	1.5mA - 0.5mA	0.373	1.000	0.013
	1.5mA - sham	8.466	< 0.001***	0.301
	1.0mA - 0.5mA	0.533	1.000	0.019
	1.0mA - sham	8.650	< 0.001***	0.307
	0.5mA - sham	8.069	< 0.001***	0.288
Beta	1.5mA - 1.0mA	-3.716	0.001**	-0.130
	1.5mA - 0.5mA	-1.887	0.355	-0.067
	1.5mA - sham	8.040	< 0.001***	0.286
	1.0mA - 0.5mA	1.810	0.421	0.064
	1.0mA - sham	11.741	< 0.001***	0.416
	0.5mA - sham	9.880	< 0.001***	0.353
Gamma	1.5mA - 1.0mA	-9.750	< 0.001***	-0.342
	1.5mA - 0.5mA	-7.206	< 0.001***	-0.254
	1.5mA - sham	3.028	0.015*	0.108
	1.0mA - 0.5mA	2.489	0.077	0.088
	1.0mA - sham	12.681	< 0.001***	0.450
	0.5mA - sham	10.146	< 0.001***	0.362

Note. * $p < 0.05$; ** $p < 0.01$; *** $p < 0.001$.

2.2 Effects of beta tACS on the resting-state ImCoh of the theta, alpha, and gamma bands

The LMM analysis of resting-state ImCoh found a higher-order four-way INTENSITY × TIME × FREQUENCY × REGION interaction (χ^2 (18, N = 21) = 107.64, p < 0.001). Post-hoc analyses of resting-state beta ImCoh were presented in the main manuscript. Here, we focus on describing the post-hoc analyses of resting-state theta, alpha, and gamma ImCoh.

2.2.1 Results

2.2.1.1 Theta ImCoh

As shown in Supplementary Figure 2, increases in theta ImCoh were observed following sham, 0.5 mA, and 1.5 mA stimulation, from pre to $post_1$ ($|zs| \geq 3.893$, $ps < 0.001$, $|ds| \geq 0.137$) and from pre to $post_2$ ($|zs| \geq 4.232$, $ps < 0.001$, $|ds| \geq 0.149$). Most stimulation intensities did not show a significant change from $post_1$ to $post_2$ ($|zs| \leq 1.989$, $ps \geq 0.140$, $|ds| \leq 0.069$), with only sham stimulation showing a significant change – a decrease in theta power ($z = 3.340$, $p = 0.003$, $d = 0.117$). The increases in theta ImCoh were unlikely due to tACS, as the extent of changes following real stimulation were not greater than the changes following sham stimulation ($|zs| \leq 2.534$, $ps \geq 0.203$, $|ds| \leq 0.181$). Notably, 1.0 mA stimulation did not evoke any change in theta ImCoh ($|zs| \leq 1.989$, $ps \geq 0.140$, $|ds| \leq 0.069$).

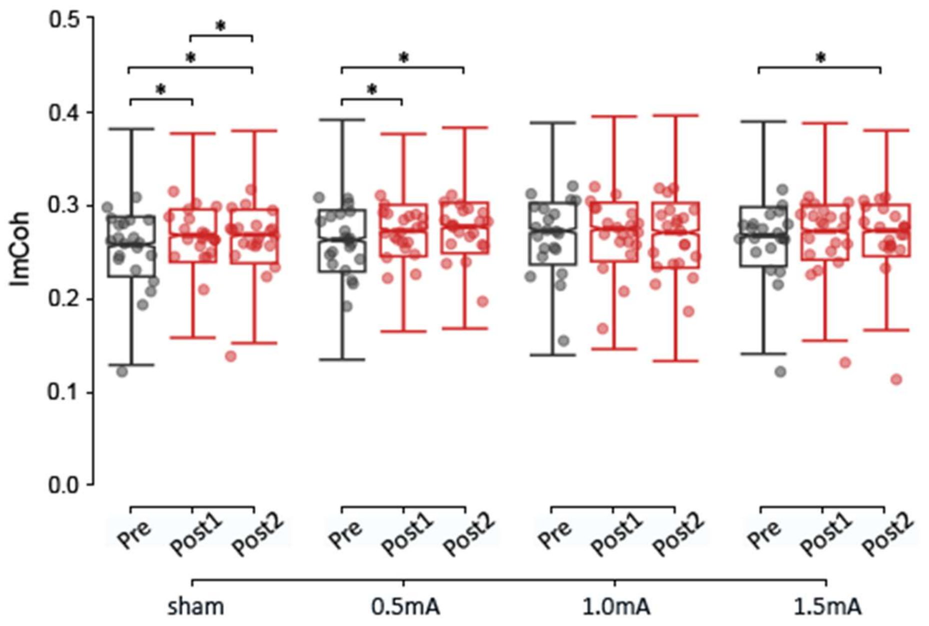

Supplementary Figure 2. Resting-state theta ImCoh for each time-point and stimulation intensity. ImCoh values have been baseline-normalised to the period of -2000 to -1000 ms. * = significant change between time-points at α = 0.05. Data points reflect participant averages. The height of the notches reflect the median +/- 1.57 x IQR/sqrt(n) where IQR is the interquartile range defined by the 25th and 75th percentiles and n is the number of data points.

2.2.1.2 Alpha ImCoh

As shown in Supplementary Figure 3, increases in alpha ImCoh were observed following sham, 0.5 mA, and 1.0 mA stimulation, from pre to $post_1$ ($|zs| \geq 5.495$, $ps < 0.001$, $|ds| \geq 0.190$) and from pre to $post_2$ ($|zs| \geq 5.071$, $ps < 0.001$, $|ds| \geq 0.178$). None of the stimulation intensities showed a significant change in alpha ImCoh from $post_1$ to $post_2$ ($|zs| \leq 0.835$, $ps = 1.000$, $|ds| \leq 0.029$). The increases in alpha ImCoh were unlikely due to tACS, as the extent of changes following real stimulation were not greater than the changes following sham stimulation ($|zs| \leq 1.596$, $ps = 1.000$, $|ds| \leq 0.114$). Notably, 1.5 mA stimulation did not evoke any change in alpha ImCoh ($|zs| \leq 1.914$, $ps \geq 0.167$, $|ds| \leq 0.067$).

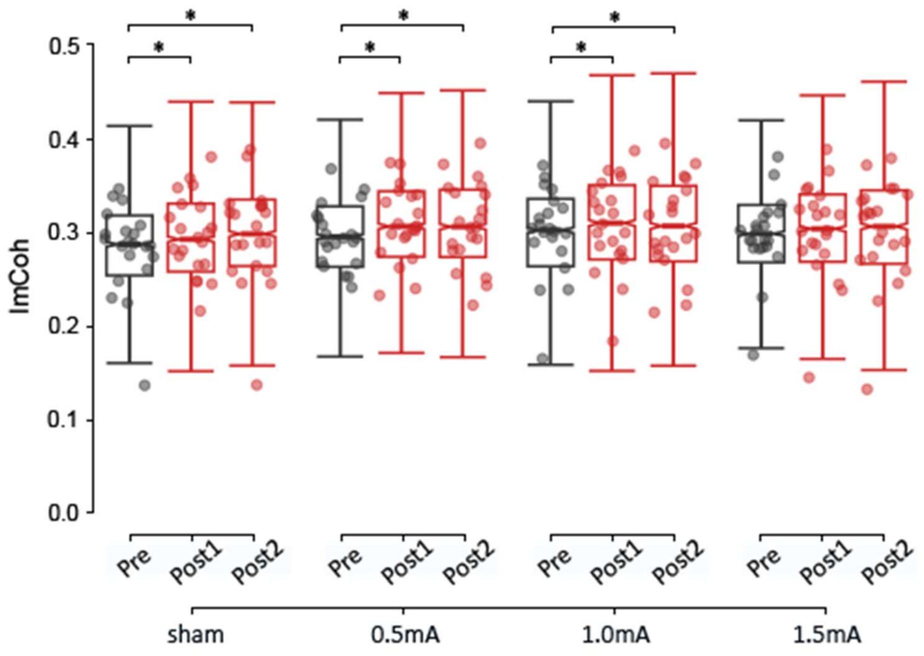

Supplementary Figure 3. Resting-state alpha ImCoh for each time-point and stimulation intensity. ImCoh values have been baseline-normalised to the period of -2000 to -1000 ms. * = significant change between time-points at $\alpha = 0.05$. Data points reflect participant averages. The height of the notches reflect the median +/- 1.57 x IQR/sqrt(n) where IQR is the interquartile range defined by the 25th and 75th percentiles and n is the number of data points.

2.2.1.3 Gamma ImCoh

As shown in Supplementary Figure 4, decreases in gamma ImCoh were observed from pre to $post_1$ following all real simulation intensities ($|zs| \geq 3.715$, $ps < 0.001$, $|ds| \geq 0.131$). At $post_2$, gamma ImCoh remained suppressed (relative to baseline) following 1.0 mA and 1.5 mA stimulation ($|zs| \geq 2.914$, $ps \leq 0.011$, $|ds| \geq 0.102$), but not 0.5 mA stimulation ($z = 0.500$, $p = 1.000$, $d = 0.018$). Relative to baseline, sham stimulation did not induce a significant change in gamma ImCoh ($|zs| \leq 1.911$, $ps \geq 0.168$, $|ds| \leq 0.068$). However, from $post_1$ to $post_2$, a decrease in gamma ImCoh was observed following sham stimulation ($z = 2.600$, $p = 0.028$, $d = 0.091$), while ImCoh began to return to baseline levels following 0.5 mA and 1.0 mA stimulation ($|zs| \geq 7.805$, $ps < 0.001$, $|ds| \geq 0.276$) but not 1.5 mA stimulation ($z = 0.027$, $p = 1.000$, $d = 0.001$).

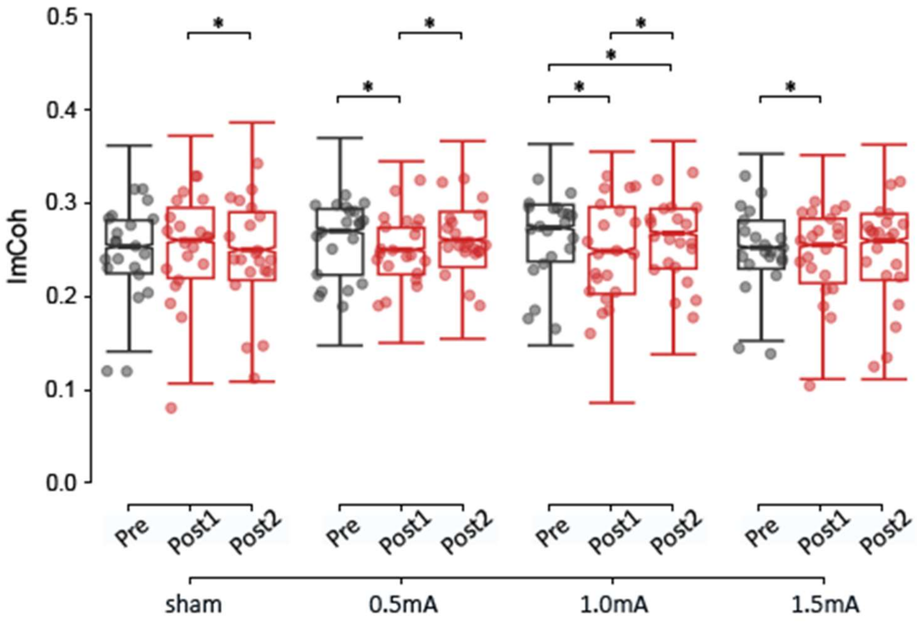

Supplementary Figure 4. Resting-state gamma ImCoh for each time-point and stimulation intensity. ImCoh values have been baseline-normalised to the period of -2000 to -1000 ms. * = significant change between time-points at α = 0.05. Data points reflect participant averages. The height of the notches reflect the median +/- 1.57 x IQR/sqrt(n) where IQR is the interquartile range defined by the 25th and 75th percentiles and n is the number of data points.

2.3 Baseline differences in event-related ImCoh between stimulation intensities

In the main manuscript, separate LMM analyses were performed for each movement period to examine the effect of tACS on the event-related ImCoh. Model estimates were obtained for the fixed effects of INTENSITY, TIME, and FREQUENCY. Results revealed no significant differences in ImCoh the stimulation intensities at baseline for any movement period. The results for the pre-movement, movement, and post-movement periods have been reported in Supplementary Table 3, Supplementary Table 4, and Supplementary Table 5, respectively. As the highest level of significant interaction was a two-way interaction between INTENSITY and TIME, the baseline differences in broadband (4 – 90 Hz) ImCoh have been reported.

Supplementary Table 3. Baseline differences between stimulation intensities in ImCoh of the pre-movement period.

**Intensity comparisons**	***z***	***p***	**Effect size (*d*)**
1.5mA - 1.0mA	-1.193	1.000	-0.029
1.5mA - 0.5mA	-0.120	1.000	-0.003
1.5mA - sham	-2.068	0.232	-0.050
1.0mA - 0.5mA	1.083	1.000	0.026
1.0mA - sham	-0.863	1.000	-0.021
0.5mA - sham	-1.962	0.299	-0.047

Supplementary Table 4. Baseline differences between stimulation intensities in ImCoh of the movement period.

**Intensity comparisons**	***z***	***p***	**Effect size (*d*)**
1.5mA - 1.0mA	1.380	1.000	0.034
1.5mA - 0.5mA	0.710	1.000	0.017
1.5mA - sham	-0.877	1.000	-0.021
1.0mA - 0.5mA	-0.680	1.000	-0.016
1.0mA - sham	-2.268	0.140	-0.055
0.5mA - sham	-1.600	0.658	-0.038

Supplementary Table 5. Baseline differences between stimulation intensities in ImCoh of the post-movement period.

**Intensity comparisons**	***z***	***p***	**Effect size (*d*)**
1.5mA - 1.0mA	0.946	1.000	0.023
1.5mA - 0.5mA	1.820	0.413	0.044
1.5mA - sham	-0.544	1.000	-0.013
1.0mA - 0.5mA	0.865	1.000	0.021
1.0mA - sham	-1.498	0.805	-0.036
0.5mA - sham	-2.383	0.103	-0.057
